# Supplementary material for: Contrasting behaviour under pressure reveals the reasons for pyramidalization in tris(amido)uranium(III) and tris(arylthiolate) uranium(III) molecules
Source: Nat Commun. 2022 Jul 7;13:3931. doi: 10.1038/s41467-022-31550-7 (PMC9262880; doi:10.1038/s41467-022-31550-7)
Supplement: Supplementary file 1 — Supplementary Information [file 41467_2022_31550_MOESM1_ESM.pdf]

Supplementary Information: Contrasting behaviour under pressure reveals the reasons for pyramidalization in tris(amido)uranium(III) and tris(arylthiolate) uranium(III) molecules

Amy N. Price,[1, 2] Victoria Berryman,[3] Tatsumi Ochiai,[1] Jacob J. Shephard,[1] Simon Parsons,*[1] Nikolas Kaltsoyannis,*[3] Polly L. Arnold*[1, 2]

Affiliations

1. EaStCHEM School of Chemistry and The Centre for Science at Extreme Conditions. The University of Edinburgh, King's Buildings, Edinburgh, UK, EH9 3FJ. 2. University of California, Berkeley and Lawrence Berkeley National Laboratory, Berkeley, California CA 94720, US. 3. Department of Chemistry, The University of Manchester, Oxford Road, Manchester, UK, M13 9PL.

Supplementary Figures S-1

Supplementary Tables S-2
Crystallographic Information Table for **UN3** S-2
Crystallographic Information Table for **US3** S-5
Supplementary Methods S-7
Preparation of **UN3** S-7
Preparation of **US3** S-7

Supplementary Notes S-7
Structure of **UN3** S-7
Structure of **US3** S-10
Volume change of **UN3** and **US3** with pressure S-18
Computational details and analysis S-18
Optimised cartesian coordinates S-20

Supplementary References S-23

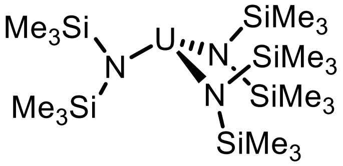

UN3

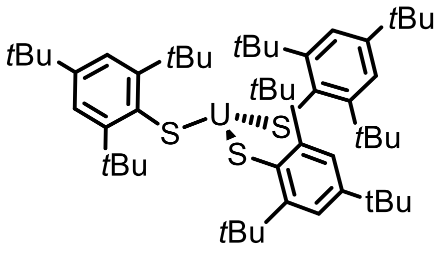

US3

Supplementary Figure 1. UN3 and US3. **UN3** = uranium tris(hexamethyldisilylamide)), and **US3** = uranium tris(1-thio-2,4,6-tri-tert-butylphenyl.

Supplementary Tables

Supplementary Table 1. Crystal and Refinement Data for UN3. Crystallographic information for **UN3** ($U(N(SiMe_3)_2)_3$) at various pressures.

For all structures: trigonal, *P*-31*c*, *Z* = 2, chemical formula: $C_{24}H_{66}N_3Si_6U$, *M* = 803.35. H-atom parameters were not refined.

Pressure (GPa)	0.00	0.87	2.37	2.62
Crystal data				
Temperature (K)	270	293	293	293
a, *c* (Å)	16.3262 (3), 8.2831 (2)	15.8983 (7), 8.1365 (4)	15.3562 (5), 7.9556 (10)	15.2929 (5), 7.9306 (10)
V ($Å^3$)	1912.03 (8)	1781.02 (17)	1624.7 (2)	1606.3 (2)
Radiation type	Mo $K\alpha$	Mo $K\alpha$	Synchrotron, λ = 0.48590 Å	Synchrotron, λ = 0.48590 Å
μ (mm^{-1})	4.45	4.78	3.09	3.12
Crystal size (mm)	0.30 × 0.20 × 0.10	0.20 × 0.15 × 0.10	0.20 × 0.15 × 0.10	0.20 × 0.15 × 0.10
Data collection				
Diffractometer	Bruker Apex2	Bruker Apex2	Synchrotron	Synchrotron
Absorption correction	Multi-scan *SADABS* (Siemens, 1996)	Multi-scan *SADABS* (Siemens, 1996)	Multi-scan *AIMLESS* (Evans and Murshudov, 2013)	Multi-scan *AIMLESS* (Evans and Murshudov, 2013)
T_{min}, T_{max}	0.49, 0.64	0.49, 0.62	0.997, 1	0.922, 1.0
No. of measured, independent and observed [$I > 2.0\sigma(I)$] reflections	22245, 1656, 1326	5580, 827, 542	5569, 887, 554	5429, 879, 403
R_{int}	0.030	0.060	0.090	0.080
$(\sin \theta/\lambda)_{max}$ ($Å^{-1}$)	0.680	0.637	0.588	0.588
Refinement				
$R[F^2 > 2\sigma(F^2)]$, $wR(F^2)$, S	0.032, 0.034, 1.04	0.063, 0.043, 1.16	0.053, 0.044, 1.00	0.047, 0.017, 0.99
No. of reflections	1326	542	554	403
No. of parameters	53	52	54	54
No. of restraints	27	37	33	34
H-atom treatment	H-atom parameters not refined	H-atom parameters not refined	H-atom parameters not refined	H-atom parameters constrained
$\Delta\rho_{max}$, $\Delta\rho_{min}$ (e $Å^{-3}$)	0.69, -0.44	0.62, -0.57	1.28, -0.61	0.76, -1.56

Pressure (GPa)	2.83	3.10	3.37	3.53
Crystal data				
Chemical formula	$C_{24}H_{39}N_3Si_6U$	$C_{24}H_{39}N_3Si_6U$	$C_{24}H_{39}N_3Si_6U$	$C_{24}H_{39}N_3Si_6U$
M_r	776.14	776.14	776.14	776.14
Temperature (K)	293	293	293	293
a, *c* (Å)	15.2359 (6), 7.9097 (11)	15.1790 (6), 7.8874 (13)	15.1212 (6), 7.8666 (13)	15.0891 (6), 7.8515 (12)
V (Å^3)	1590.1 (2)	1573.8 (3)	1557.7 (3)	1548.1 (3)
Radiation type	Synchrotron, λ = 0.48590 Å	Synchrotron, λ = 0.48590 Å	Synchrotron, λ = 0.48590 Å	Synchrotron, λ = 0.48590 Å
μ (mm^{-1})	3.15	3.19	3.22	3.24
Crystal size (mm)	0.20 × 0.15 × 0.10	0.20 × 0.15 × 0.10	0.20 × 0.15 × 0.10	0.20 × 0.15 × 0.10
Data collection				
Diffractometer	Synchrotron	Synchrotron	Synchrotron	Synchrotron
Absorption correction	Multi-scan *AIMLESS* (Evans and Murshudov, 2013)	Multi-scan *AIMLESS* (Evans and Murshudov, 2013)	Multi-scan *AIMLESS* (Evans and Murshudov, 2013)	Multi-scan *AIMLESS* (Evans and Murshudov, 2013)
T_{min}, T_{max}	0.926, 1.0	0.997, 1.0	0.995, 1.0	0.930, 1.0
No. of measured, independent and observed [$I > 2.0\sigma(I)$] reflections	5286, 869, 434	5358, 850, 488	5288, 837, 470	5189, 836, 417
R_{int}	0.075	0.100	0.094	0.075
$(\sin \theta/\lambda)_{max}$ (Å^{-1})	0.588	0.588	0.588	0.588
Refinement				
$R[F^2 > 2\sigma(F^2)]$, $wR(F^2)$, S	0.047, 0.017, 1.00	0.055, 0.043, 1.55	0.055, 0.042, 0.99	0.046, 0.017, 0.98
No. of reflections	434	488	470	417
No. of parameters	54	54	54	54
No. of restraints	34	33	34	34
H-atom treatment	H-atom parameters not refined	H-atom parameters not refined	H-atom parameters not refined	H-atom parameters not refined
$\Delta\rho_{max}$, $\Delta\rho_{min}$ (e Å^{-3})	0.53, -0.56	1.22, -0.61	1.23, -0.60	0.54, -0.91

Pressure (GPa)	3.74	4.09
Crystal data		
Chemical formula	$C_{24}H_{39}N_3Si_6U$	$C_{24}H_{39}N_3Si_6U$
M_r	776.14	776.14
Temperature (K)	293	293
a, *c* (Å)	15.0508 (6), 7.8334 (12)	14.9766 (8), 7.8102 (17)
V ($Å^3$)	1536.7 (3)	1517.1 (3)
Radiation type	Synchrotron, λ = 0.48590 Å	Synchrotron, λ = 0.48590 Å
μ (mm^{-1})	3.26	3.31
Crystal size (mm)	0.20 × 0.15 × 0.10	0.20 × 0.15 × 0.10
Data collection		
Diffractometer	Synchrotron	Synchrotron
Absorption correction	Multi-scan *AIMLESS* (Evans and Murshudov, 2013)	Multi-scan *AIMLESS* (Evans and Murshudov, 2013)
T_{min}, T_{max}	0.917, 1.0	0.956, 1.0
No. of measured, independent and observed [$I > 2.0\sigma(I)$] reflections	5198, 835, 360	4927, 828, 385
R_{int}	0.078	0.086
$(\sin \theta/\lambda)_{max}$ ($Å^{-1}$)	0.587	0.588
Refinement		
$R[F^2 > 2\sigma(F^2)]$, $wR(F^2)$, S	0.046, 0.014, 1.00	0.049, 0.018, 1.00
No. of reflections	360	385
No. of parameters	54	54
No. of restraints	34	34
H-atom treatment	H-atom parameters not refined	H-atom parameters not refined
$\Delta\rho_{max}$, $\Delta\rho_{min}$ (e $Å^{-3}$)	0.46, -0.77	0.80, -1.29

Supplementary Table 2. Crystal and Refinement Data for US3. Crystallographic information for **US3** (uranium tris(1-thio-2,4,6-tri-tert-butylbenzene) at varying pressures.

For all structures: $C_{54}H_{87}S_3U$, M_r = 1070.51, monoclinic, $P2_1/n$, Z = 4. Experiments were carried out at 293 K. H-atom parameters were not refined.

Pressure (GPa)	0.00	0.62	3.52	3.75
Crystal data				
a, *b*, *c* (Å)	10.2087 (4), 37.9204 (16), 14.4517 (6)	9.8723 (12), 36.678 (5), 14.1390 (8)	9.3536 (2), 34.8634 (11), 13.5316 (5)	9.3013 (2), 34.6630 (13), 13.4571 (5)
β (°)	99.806 (2)	99.749 (7)	98.6070 (19)	98.504 (2)
V (Å^3)	5512.8 (4)	5045.8 (10)	4362.9 (2)	4291.0 (2)
Radiation type	Mo *K*α	Synchrotron, λ = 0.68890 Å	Synchrotron, λ = 0.48590 Å	Synchrotron, λ = 0.48590 Å
μ (mm^{-1})	3.09	3.10	2.31	2.35
Crystal size (mm)	0.40 × 0.30 × 0.20	0.20 × 0.15 × 0.10	0.20 × 0.15 × 0.10	0.20 × 0.15 × 0.10
Data collection				
Diffractometer	Bruker Apex2	Synchrotron	Synchrotron	Synchrotron
Absorption correction	Multi-scan *SADABS* (Siemens, 1996)	Multi-scan *AIMLESS* (Evans and Murshudov, 2013)	Multi-scan *AIMLESS* (Evans and Murshudov, 2013)	Multi-scan *AIMLESS* (Evans and Murshudov, 2013)
T_{min}, T_{max}	0.47, 0.54	0.995, 1	0.998, 1.0	0.997, 1.0
No. of measured, independent and observed [$I > 2.0\sigma(I)$] reflections	47262, 13012, 7615	8189, 4112, 2426	27369, 8208, 6079	27077, 8005, 6106
R_{int}	0.095	0.121	0.071	0.094
$(\sin \theta/\lambda)_{max}$ (Å^{-1})	0.667	0.647	0.797	0.800
Refinement				
$R[F^2 > 2\sigma(F^2)]$, $wR(F^2)$, S	0.059, 0.033, 1.00	0.098, 0.134, 1.00	0.050, 0.051, 1.00	0.051, 0.058, 1.00
No. of reflections	7615	2426	6079	6106
No. of parameters	523	253	523	523
No. of restraints	171	93	264	264
$\Delta\rho_{max}$, $\Delta\rho_{min}$ (e Å^{-3})	2.11, -2.20	2.01, -1.34	2.52, -1.74	3.32, -1.57

Pressure (GPa)	4.58	4.91	5.25
Crystal data			
a, *b*, *c* (Å)	9.2295 (2), 34.3321 (15), 13.3486 (3)	9.2040 (2), 34.2000 (18), 13.3109 (4)	9.1770 (2), 34.0712 (17), 13.2711 (4)
β (°)	98.4049 (17)	98.3630 (18)	98.3239 (17)
V (Å^3)	4184.3 (2)	4145.4 (3)	4105.8 (3)
Radiation type	Synchrotron, λ = 0.48590 Å	Synchrotron, λ = 0.48590 Å	Synchrotron, λ = 0.48590 Å
μ (mm^{-1})	2.41	2.43	2.45
Crystal size (mm)	0.20 × 0.15 × 0.10	0.20 × 0.15 × 0.10	0.20 × 0.15 × 0.10
Data collection			
Diffractometer	Synchrotron	Synchrotron	Synchrotron
Absorption correction	Multi-scan *AIMLESS* (Evans and Murshudov, 2013)	Multi-scan *AIMLESS* (Evans and Murshudov, 2013)	Multi-scan *AIMLESS* (Evans and Murshudov, 2013)
T_{min}, T_{max}	0.994, 1.0	0.994, 1.0	0.993, 1.0
No. of measured, independent and observed [$I > 2.0\sigma(I)$] reflections	26060, 7613, 5440	25883, 7691, 5304	25643, 7707, 5432
R_{int}	0.056	0.062	0.063
$(\sin \theta/\lambda)_{max}$ (Å^{-1})	0.796	0.799	0.796
Refinement			
$R[F^2 > 2\sigma(F^2)]$, $wR(F^2)$, S	0.043, 0.039, 1.00	0.045, 0.040, 1.00	0.049, 0.046, 1.00
No. of reflections	5440	5304	5432
No. of parameters	523	523	523
No. of restraints	264	264	264
$\Delta\rho_{max}$, $\Delta\rho_{min}$ (e Å^{-3})	3.21, -1.40	3.39, -1.21	3.72, -1.38

Supplementary Methods

General Information

All manipulations were carried out under a dry, oxygen-free (less than 0.5 ppm O_2) atmosphere of dinitrogen using standard glovebox techniques. Glassware, Fisherbrand 1.2 µm retention glass microfiber filters were dried in an oven at 160 °C for at least 12 hours prior to use. Celite was dried under vacuum at 100 °C for 18 hours prior to use. All solvents were purchased from Sigma Aldrich or Fisher Scientific. Toluene, THF, pentane and hexane were collected from a Vac Atmospheres solvent tower drying system after cycling through a column of molecular sieves for a minimum of 12 hours, degassed and stored in ampoules containing activated 4 Å molecular sieves prior to use. C_6D_6 was heated under reflux over potassium for 24 hours, freeze-pump-thaw degassed and collected by trap-to-trap distillation and stored under dinitrogen. All commercial solid reagents were used as purchased.

^{1}H NMR spectra were collected on an AVA 500 Bruker instrument and are referenced using the residual 1 H resonances in deuterated solvents. UI_3(1,4-dioxane)$_{1.5}$ and HSAr (HSAr = HS-2,4,6-tris-*tert*-butylbenzene) were prepared according to literature procedures.[1][2] **UN3** and **US3** were prepared according to literature procedure with minor modifications.[3][4]

UN3

A 100 mL flask was charged with NaN (Na(NSiMe$_3$)$_2$) (0.73 g, 4.0 mmol), UI_3(1,4-dioxane)$_{1.5}$ (1.0 g, 1.3 mmol) in thf (45 mL). After stirring for one hour, the resulting cloudy purple suspension was filtered on a medium porosity frit with a celite pad. The volatiles were removed *in vacuo* and the resulting dark purple-red residue was extracted into pentane (50 mL), followed by filtration on a medium porosity frit with a celite pad. The volatiles were removed *in vacuo* from the filtrate, yielding **UN3** as a purple-red powder (0.70 g, 0.97 mmol, 73% yield). Crystals suitable for single crystal X-ray diffraction were grown from a concentrated solution of **UN3** in hexane at -35°C. The ^{1}H-NMR spectrum is consistent with that previously reported.[3] ^{1}H-NMR (C_6D_6, 300 K): δ -11.4 (s, 54H, Si<u>*Me*</u>$_3$).

US3

UN3 (33 mg 0.048 mmol) and HSAr (35 mg 0.13 mmol) were charged to a vial with 5 mL of cyclohexane. The resulting dark solution was stirred for two hours at room temperature. After filtration on a pipette filter (glass fibre filter with a celite pad), the volatiles were removed from the filtrate *in vacuo*. The resulting black residue was dissolved in a minimum of toluene and stored at -35°C, yielding black crystals (19 mg, 0.018 mmol, 43% yield upon filtration) after one week. These crystals were of suitable quality for single crystal X-ray diffraction analysis.

Supplementary Notes

Additional discussion of the structure of UN3 at both ambient and high pressure

a **b**

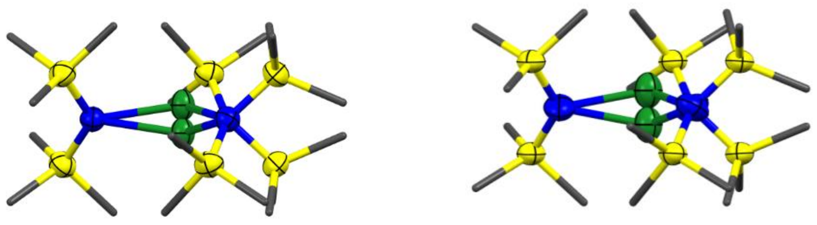

Supplementary Figure 2: Structure of UN3 at ambient and high pressure. a) Structure of **UN3** at ambient pressure and **b)** at 4.09 GPa, showing the disorder of U atoms above and below the planes of the N atoms. Green = U; blue = N; gold = Si; grey = C. **UN3** = U(N(SiMe$_3$)$_2$)$_3$

Diffraction data were collected at ambient pressure at 270 K in order to protect the crystal from the air in a film of oil while maintaining a steady position on its mount. Data sets at high pressure were collected in a Merrill-Bassett diamond anvil cell using Boehler-Almax cut diamonds, a tungsten gasket and ruby as a pressure marker. The hydrostatic medium at above 2 GPa was a 1:1 mixture of pentane and isopentane. The sample was soluble in this

medium below 2 GPa, but a data set was collected at 0.87 GPa using Fluorinert (FC70) as the medium. Fluorinert has a hydrostatic limit of ~1 GPa, and so no data could be collected between 1 and 2.37 GPa. The maximum pressure reached was 4.09 GPa. Data at ambient pressure and 0.87 GPa were collected using MoKα radiation, those at higher pressures were collected at Diamond Light Source on Beamline I19 (Experimental Hutch 2) with X-rays of wavelength 0.4859 Å. Shaded regions of the detector were omitted during integration of the diffraction images, and a multi-scan correction was used to account for absorption, gasket shading and other systematic errors.

The crystal structure of **UN3** is in space group *P*-31*c* with two formula units per unit cell. The structure at ambient pressure was solved using direct methods (SIR92) and refined against |*F*| using CRYSTALS. Explicit distance and angle restraints (taken from CSD refcode CYCHEX) were applied to a molecule of cyclohexane of solvation which is disordered about a -3 site. The complex occupies a 3.2 site. The N-atoms lie on ..2-fold axes so that the N" groups have two-fold symmetry. The three-fold axis passes through the U atom, which resides just 0.352(1) Å above the intersection of the 3.. and ..2 elements, generating two closely-located U-sites each with 50% occupancy and trigonal pyramidal coordination geometry (Supplementary Figure 2). Refinement of structures at higher pressure were started from the coordinates obtained at the previous pressure point. The bond distances and angles within the ligands and solvate were restrained to values seen at ambient pressure. The co-crystallized molecule of cyclohexane begins to show signs of ordering at 0.87 GPa and is ordered at 2.37 GPa.

The ambient pressure structure of **UN3** is essentially isomorphous to the previously reported **LnN3** (Ln = Tb, Sc, Sm, Yb, Ce, Nd, Dy, Er, Y) and crystallizes in the same space group with similar unit cell dimensions and packing. As in the previous report, **UN3** has unequal U-N-Si angles of 110.17(10)° and 123.60(11)° since the Si-C bond of one of each amido ligands is closer to the U center [U···C_γ 3.055(5) Å ; U···Si 3.2800(12) Å]. These can be attributed to stabilization by agostic M···Si-C_γ interactions, similar to that discussed for [U{CH($SiMe_3$)$_2$}$_3$] and **SmN3**.[5,6]

The X-ray structure of **UN3** was determined at a range of pressures up to 4.09 GPa. The 50:50 occupancy of the U positions in the structure is maintained at high pressure. With increasing pressure, the pyramidalization at the U center, with the out-of-plane distance between the uranium and the N3 plane increasing from 0.352(1) to 0.427(3) Å (0.075(3) Å increase) throughout the pressure range (Supplementary Figure 2). The distance between U and N does not change significantly with pressure (from 2.289(3) Å at ambient pressure to 2.277(6) Å at 4.09 GPa), but the most acute angle between U-N-Si decreases (from 110.17(10)° to 107.28(18)°), together with shortened U-Si distances (3.2809(8) to 3.205(2) Å) (Table S3).

	**Distance (Å)**			**Angle (°)**	
**Atoms**	**0 GPa**	**4.09 GPa**	**Atoms**	**0 GPa**	**5.25 GPa**
U1-N1	2.289(3)	2.277(6)	N1-U1-N1^{i}	117.684(9)	116.56(5)
U1-C111^{ii}	3.055(5)	2.939(7)	U1-N1-Si1^{iii}	110.17(10)	107.28(18)
U1-Si1^{iii}	3.2809(8)	3.205(2)	Si1-N1-Si1^{iii}	125.9(2)	127.6(4)
U1-H1112^{iii}	2.74	2.69			

Supplementary Table 3: Selected distances and angles in UN3 at ambient pressure and at 4.09 GPa. *i. x, x – y + 1, -z + 3/2, ii. x,1 + x − y, 3/2 − z, iii. −x + y, y, 3/2 − z.* H atom positions normalised to neutron values.

No change in orientation of the **UN3** units within the cell is observed (Supplementary Figure 3), and a 9% reduction in length in the *a* and *b* axes, and 6% reduction in the *c*-axis observed over the pressure range (Supplementary Figure 4). These changes reflect the widening of the SiNSi angle in each ligand, allowing for greater compression in the *a* and *b* axis directions, while increased pyramidalization of the **UN3** motifs prevents similar reductions in the *c*-axis length, hence the lower reduction in the unit cell *c*-axis length.

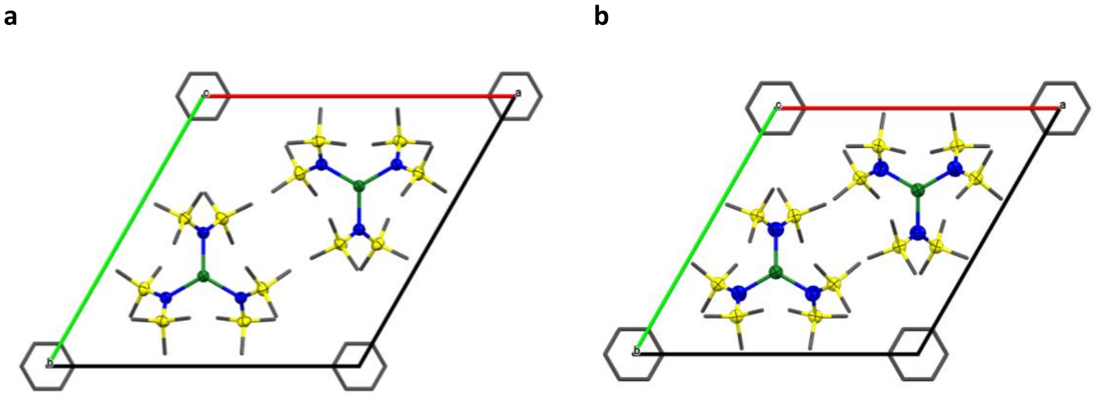

Supplementary Figure 3: Orientation of UN3 in the unit cell. **a)** at ambient pressure and **b)** at 4.09 GPa. Green = U; blue = N; gold = Si; grey = C. **UN3** = $U(N(SiMe_3)_2)_3$

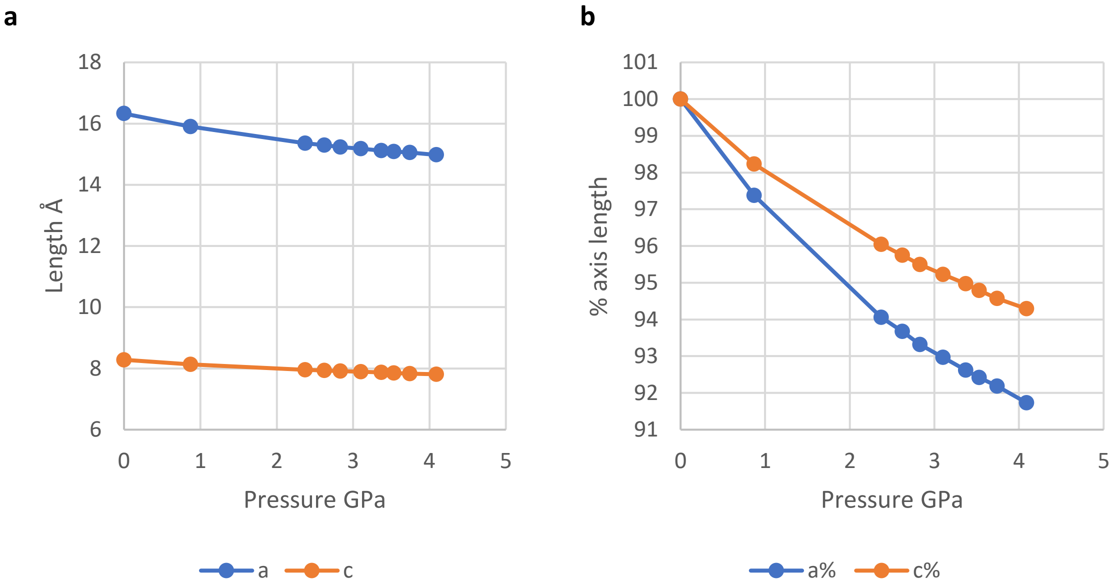

Supplementary Figure 4: Changes in the unit cell axis lengths for UN3 with pressure. a) change in unit cell lengths with pressure; **b)** percent change in unit cell axis lengths with pressure. **UN3** = $U(N(SiMe_3)_2)_3$

The unit cell volume decreases from 1912.03(8) Å^3 at ambient pressure to 1517.1(3) Å^3 at 4.09 GPa. A fit of the variation of the volume with pressure to a third order Birch-Murnaghan equation of state gave a bulk modulus at ambient pressure of 9.4(5) GPa with a pressure derivative equal to 5.4(5) (Supplementary Figure 5).[7]

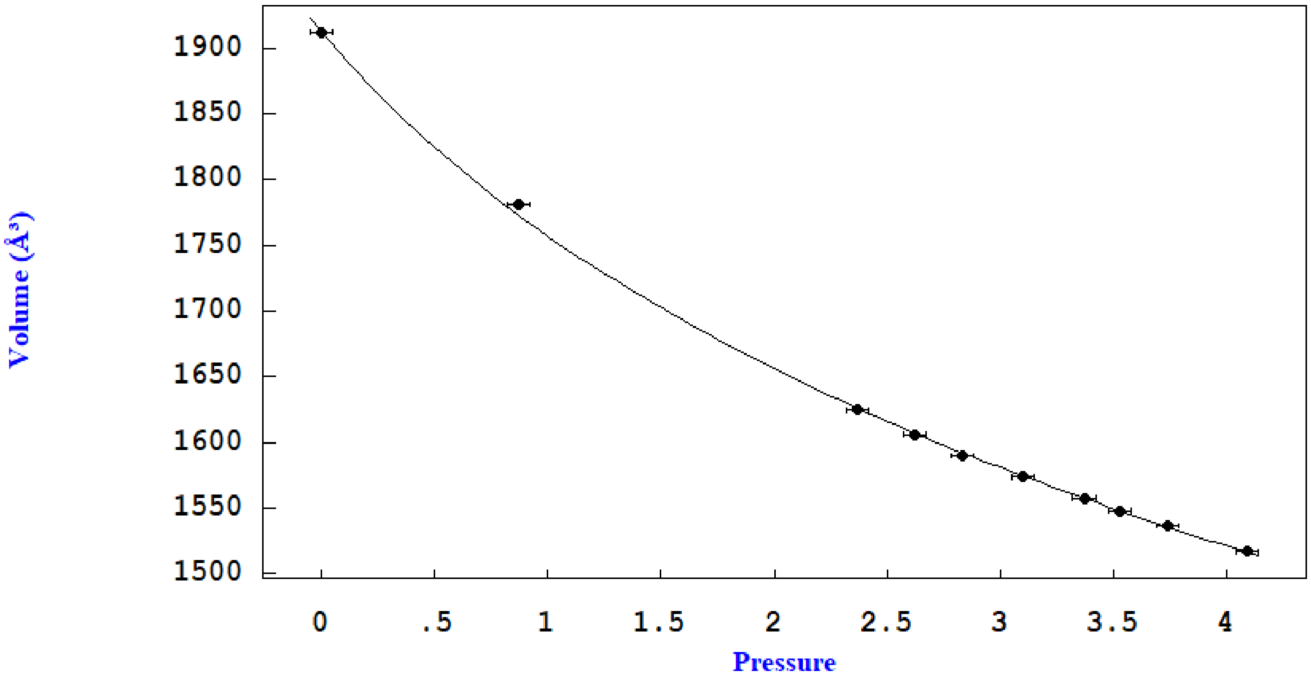

Supplementary Figure 5: Plot of the change in volume of the unit cell for UN3 with pressure (GPa). The error bars of the standard uncertainties of the unit cell volumes are shown but lie within the data symbols, pressures derived from ruby fluorescence have a typical uncertainty of 0.05 GPa. **UN3** = $U(N(SiMe_3)_2)_3$

Additional discussion of the structure of US3 at both ambient and high pressure

Data collection and refinement procedure for **US3** followed the same path as described above for **UN3**, reaching a maximum pressure of 5.25 GPa. Above 2 GPa, pressure was applied using a 1:1 mixture of pentane and isopentane as a hydrostatic medium, but similar solubility problems as were encountered with **UN3** necessitated the use of fluorinert between 0 and 1 GPa, with no data being obtainable between 1 and 2 GPa.

The structure does not contain any solvent of crystallisation, forming in space group $P2_1/n$ space group with the molecules occupying general positions. There are four molecules in the unit cell.

The U-S distances in **US3** (between 2.7057(19) and 2.7143(18) Å) are shorter than for most other reported U(III)-S distances,[8-11] apart from the recently reported tris-thiophenolate uranium complex by Meyer and co-workers which has similar U-S bond distance of 2.7083(8) Å. The $M\hat{S}C_{ipso}$ angles in **US3** are acute (78.7(2)-86.68(16)°, Table S4 below), and are somewhat more acute than those reported by Meyer and co-workers for their uranium tris-thiophenolate (92.31(9) °).[8]

The short U-C contacts in **US3** between U and the arene rings of each ligand appear to be benzylic-type interactions with the *ipso* carbons (C111, C121 and C131) and an *ortho* carbon (C126, C112 and C132) in each ring (Supplementary Figure 6, left), with U-C distances between 2.935(6)-3.364(6) Å). The alternating phenyl ring bond lengths further support this, and the effect is more prominent for U than for the Ln congeners, in agreement with the more contracted orbitals for the latter. There are also relatively short U-C(H) distances between the uranium and an ortho-*tert*-butyl CH_3 on each ligand (C1113 2.943(2) Å , C128 3.070(6) Å, C1312 3.301(8) Å).

a **b**

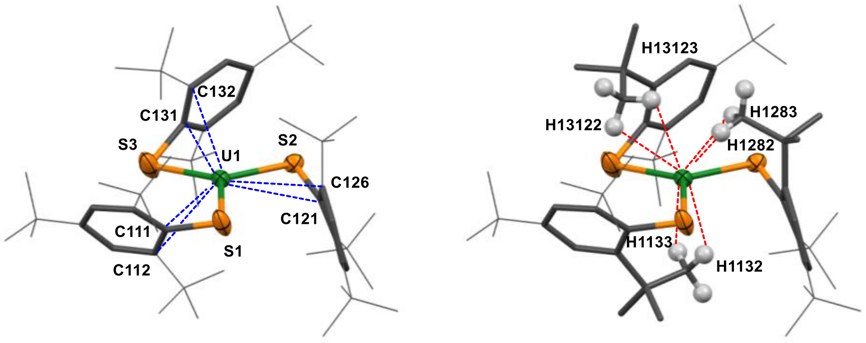

Supplementary Figure 6: Solid state structure of US3 at ambient pressure a) short C-U contacts are shown in blue **b)** ortho-^{t}Bu CH-U short contacts shown in red. **US3** = U(S-$^{t}Bu_3$-2,4,6-C_6H_2)$_3$ Atom color code: Green = U; orange = S; grey = C, white = H.

Single crystal X-ray diffraction data were collected for a single crystal of **US3** at pressures from 0.62 GPa to 5.25 GPa (Supplementary Figure 7). No phase change occurred over this pressure range. The bulk modulus of **US3** was 5.7(9) GPa with a pressure derivative of 8.5(17) (Supplementary Figure 8), which is comparable to that of other van der Waals solids e.g. $Ru_3(CO)_{12}$ is 6.6 GPa.[12]

a **b**

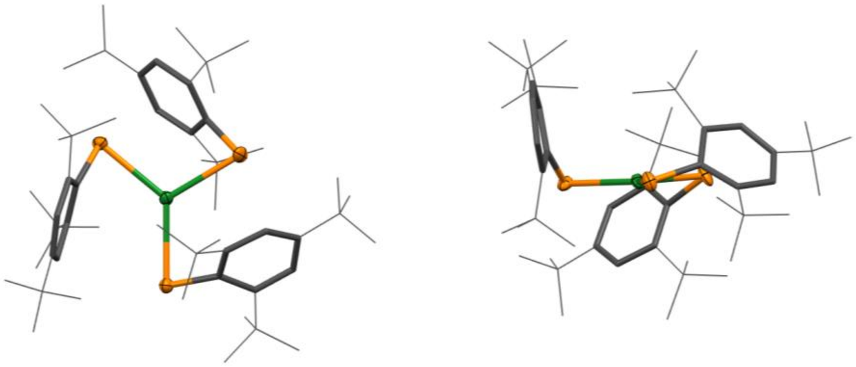

Supplementary Figure 7: Solid-state structure of US3 at 5.25 GPa a) from above the **US3** plane and **b)** along the **US3** plane. **US3** = U(S-$^{t}Bu_3$-2,4,6-C_6H_2)$_3$. Atom color code: Green = U; orange = S; grey = C.

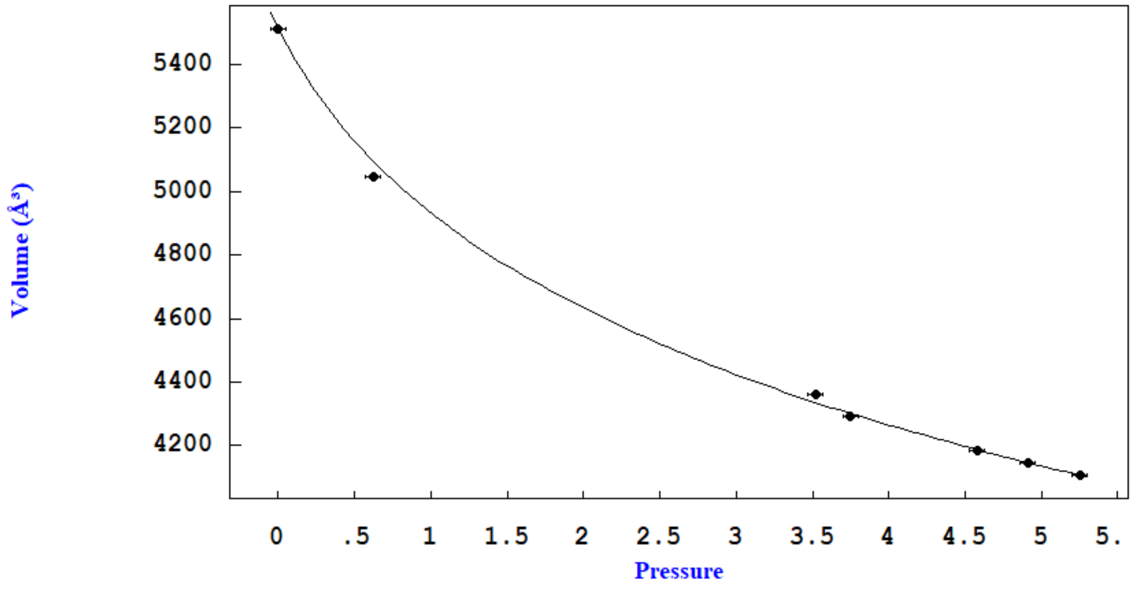

Supplementary Figure 8: Graph of the change in the unit cell volume for **US3** with pressure (GPa). The error bars of the standard uncertainties of the unit cell volumes are shown but lie within the data symbols, pressures derived from ruby fluorescence have a typical uncertainty of 0.05 GPa. **US3** = U(S-^{t}Bu$_3$-2,4,6-C_6H_2)$_3$

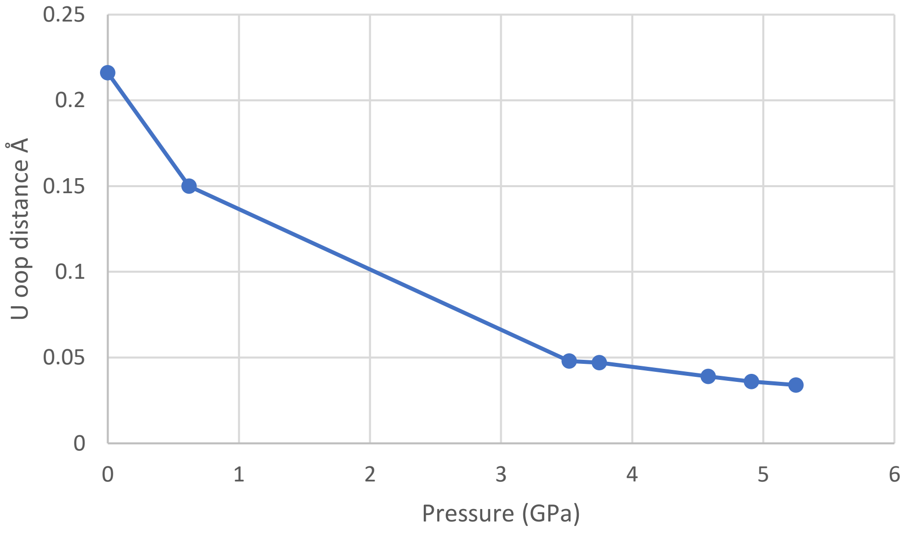

Supplementary Figure 9: Plot of the oop (out of plane) distance (Å) of U from the S3 plane in **US3** against pressure (GPa). **US3** = U(S-^{t}Bu$_3$-2,4,6-C_6H_2)$_3$.

The most notable structural change is that with increasing pressure, the uranium distance from the S3 plane decreases significantly from 0.216(1) to 0.048(1) Å by 3.52 GPa, then modestly to 0.034 Å at 5.25 GPa resulting in the essentially planar US_3 core (Supplementary Figure 7 above, Supplementary Figure 9). The short ipso and ortho U-C contacts seen in the ambient **US3** structure all contract under pressure, with the greatest change seen for ligands 1 and 3 respectively at 5.25 GPa. The shorter U-C_{ortho} contact for each ring decreases in all rings, and most for rings 1 and 3 (Table S4 below). The U-C(H) contacts with a *tert*-butyl methyl also decrease for ligands 1 and 3, while increasing in distance for ligand 2 (C1113, C1312 and C128 respectively, Table S4).

	Distance (Å)			Angle (°)	
**Atoms**	**0 GPa**	**5.25 GPa**	**Atoms**	**0 GPa**	**5.25 GPa**
U1–S1	2.7142(18)	2.742(2)	U1–S1–C111	81.66(19)	79.6(2)
U1–S2	2.7129(16)	2.7072(17)	U1–S1–C121	86.71(15)	81.63(19)
U1–S3	2.7057(19)	2.701(2)	U1–S1–C131	78.7(2)	76.2(3)
U1–C111	3.165(5)	2.984(7)			
U1–C121	2.935(6)	3.010(6)			
U1–C131	3.187(6)	2.856(9)			
U1–C112	3.364(6)	2.967(5)			
U1–C126	3.286(6)	3.233(6)			
U1–C132	3.035(6)	2.944 (7)			
U1–C1113	2.943(7)	2.887(7)			
U1–C128	3.070(6)	3.100(6)			
U1–C1312	3.301(8)	2.947(9)			

Supplementary Table 4: Selected angles and distances for the **US3** structure at ambient pressure and at 5.25 GPa. C111, C121 and C131 are the ipso-carbons of rings 1, 2 and 3 respectively, while C112, 126 and 132 are the closest ring-ortho contacts with the uranium from rings 1, 2 and 3 respectively. C1113, C128 and C1312 are the closest U-C(H) contacts with a methyl from the ortho-*tert*-butyl group of each ligand. **US3** = U(S-^{t}Bu$_3$-2,4,6-C_6H_2)$_3$.

The reduction of the length of the *a* and *b* axes, as a result of the application of pressure, is greater than that of the *c* axis from 0.6 GPa onwards (Supplementary Figure 10), with respective percent reductions of 10.2%,10.3% and 8.3% in length at 5.25 GPa compared to ambient pressure. This can be partly ascribed to rotation of the S3-plane of the **US3** molecules to be increasingly aligned with the plane of the *a* and *c*-axes (Supplementary Figure 11), this is in contrast to **UN3** for which the orientation of the **UN3** units within the cell does not change with pressure.

a **b**

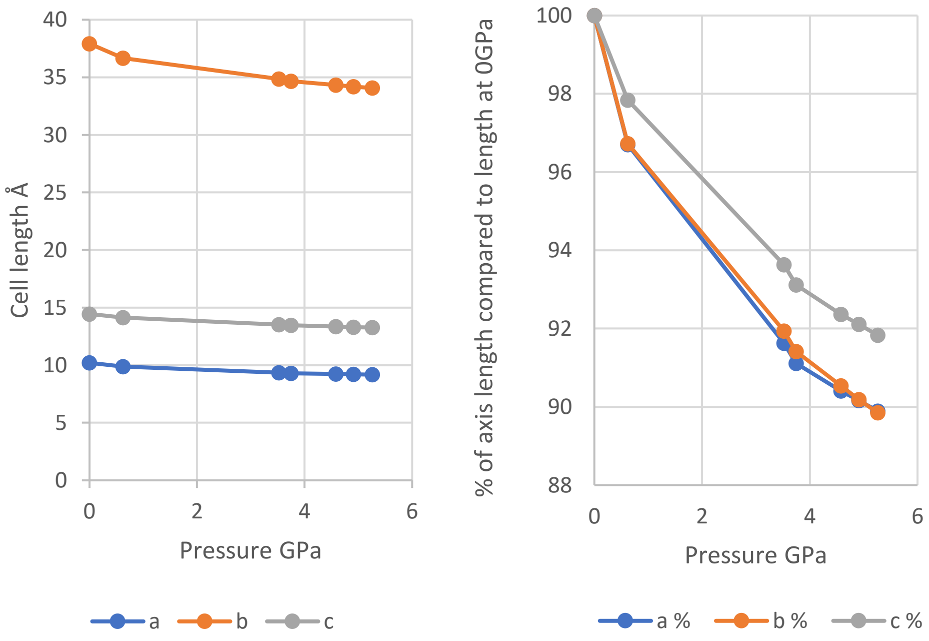

Supplementary Figure 10: Changes in the unit cell axis lengths for US3 with pressure. a) Decrease in the length of the unit cell for **US3** with pressure (Å), **b)** percent change in the axis length with increasing pressure (GPa).

a **b**

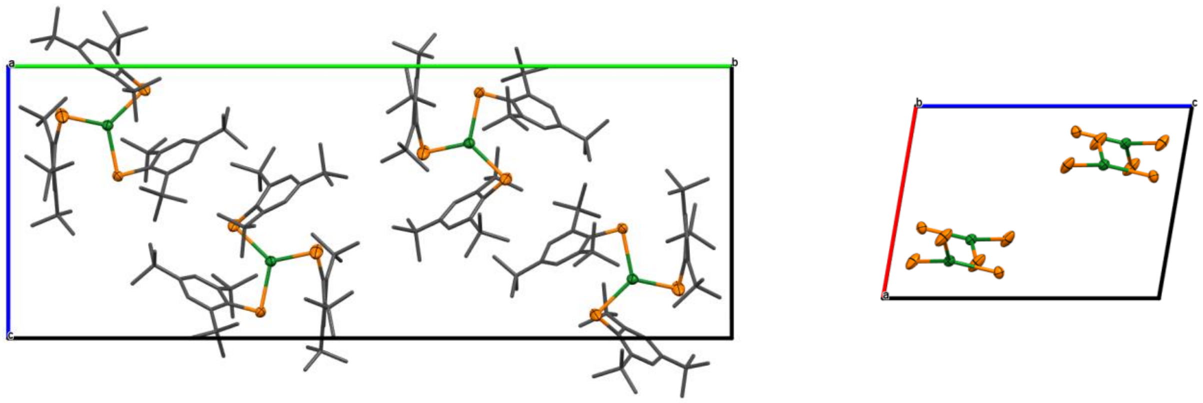

c **d**

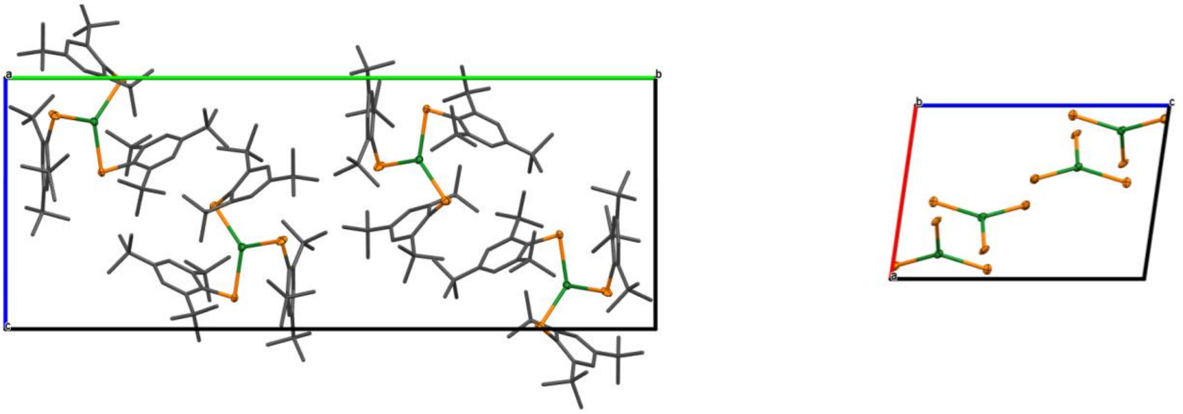

Supplementary Figure 11: Illustration of the change in the orientation of the US3 moiety within the unit cell with pressure. a) at ambient pressure looking down the a-axis **b)** at ambient pressure looking down the b-axis **c)** at 5.25 GPa looking down the a-axis and **d)** at 5.25 GPa looking down the b-axis.**US3** = U(S-^{t}Bu$_3$-2,4,6-C$_6$H$_2$)$_3$. Atom color code: Green = U; orange = S; grey = C.

Further analysis of the U-C contacts in the high-pressure structure

In the 0-3.52 GPa regime shortening of the C_{ipso}–U contacts occurs (from 2.935(6)-3.167(5) Å to 2.853(7)-3.111(6) Å), along with shortening of the shorter C_{ortho}–U, distances (from 3.187(6)-3.364(6) Å to 2.978(5)-3.339(6) Å) (Supplementary Figure 12). Beyond 3.52 GPa the changes in C_{ipso} -U distance for rings 1 and 3 are modest (from 2.994(7) and 2.853(7) Å respectively at 3.52 GPa to 2.967(7) and 2.856(9) Å respectively at 5.25 GPa), while the C_{ipso} -U distance for ring 2 remains longer than for the other two rings, but decreases consistently across the entire pressure range (3.111(6) Å at 3.52 GPa to 3.010(6) Å at 5.25 GPa. The shorter C_{ortho}-U contact for each ring decreases most again for rings 1 and 3 in the 0-3.52 GPa regime, and then with more modest reductions in distance from 3.52 GPa to 5.25 GPa (Supplementary Figure 12), while again the C_{ortho}-U distance remains longer for ring 2, but shows consistent shortening from 3.52 GPa to 5.25 GPa.

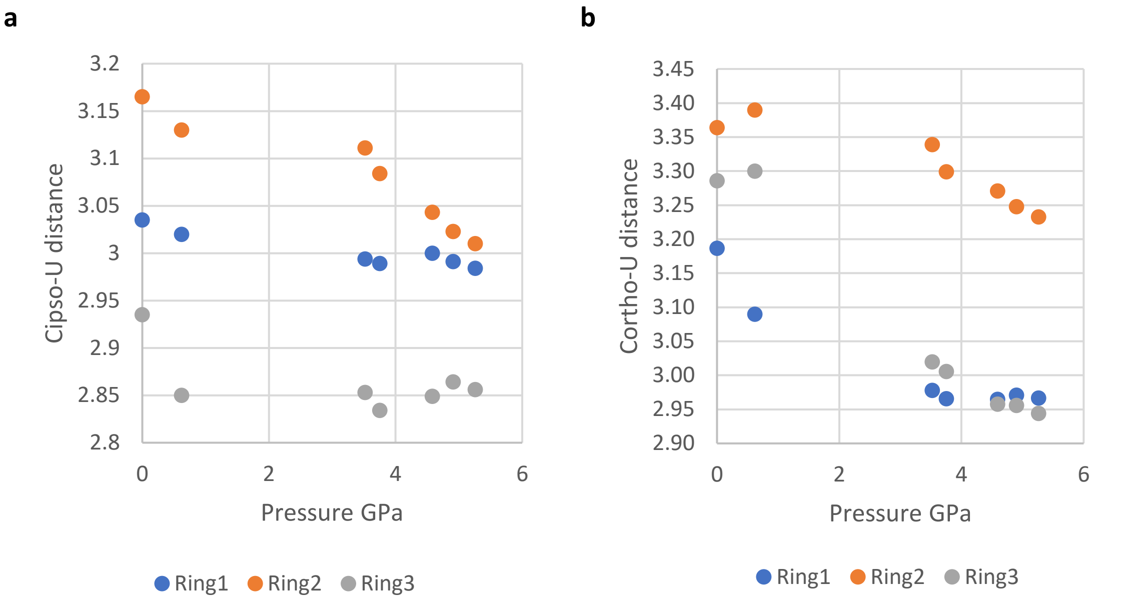

Supplementary Figure 12: **Plots of U–C_{ipso} and U–C_{ortho} distance in US3 against pressure a)** U–C_{ipso} distance (Å) and **b)** U–C_{ortho} distance (Å) (for the shortest C_{ortho} contact with U for each arene ring) against pressure (GPa). **US3** = U(S-$^{t}Bu_3$-2,4,6-C_6H_2)$_3$.

Aside from the subtle rotations in the ortho-*tert*-butyl methyls, there are small changes in the orientation of the para *tert*-butyl methyl groups on each ligand, aside from a larger rotation of 64° in the para-*tert*-butyl methyls of the ligand attached to S3, presumably to relieve intermolecular steric strain. This is accompanied by a reduction in the quaternary carbon oop distance from the phenyl ring from 0.086 Å at ambient pressure to 0.013 Å . We observed that this was accompanied by a reduction in the oop distance for the phenyl *ipso* carbon of the same ligand from 0.119 Å at ambient pressure to 0.013 Å at 5.25 GPa. A similar correlation, but in the opposite direction, is seen in ring 1, where an increase in both the C_{ipso} oop distance and the para-*tert*-butyl quaternary carbon are seen at increased pressures. A decrease for both parameters is seen for ring 3 (Supplementary Figure 13). It is thought that packing interactions for the para-*tert*-butyl groups therefore drives the degree of puckering in each ring.

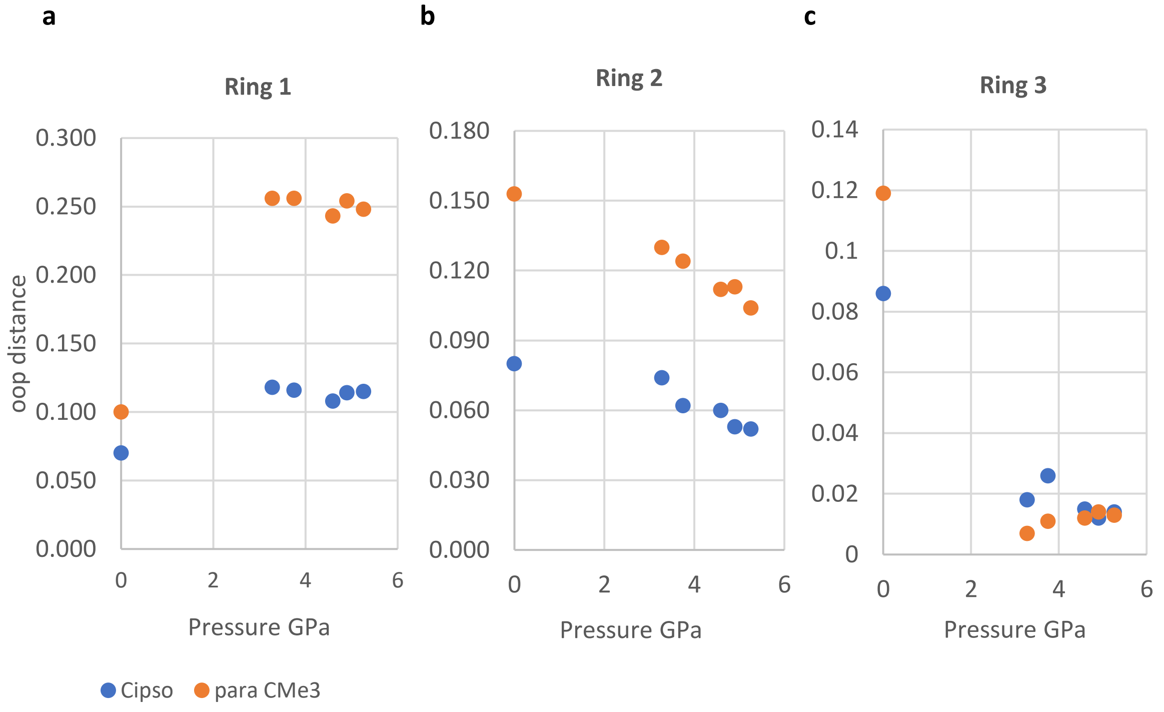

Supplementary Figure 13: The out of plane distances (Å) from the phenyl ring of the C_{ipso} carbon (blue) and the quaternary para $\underline{C}Me_3$ (orange) against pressure for each ring in US3. a) Ring 1 **b)** Ring 2 **c)** Ring 3. **US3** = U(S-$^{t}Bu_3$-2,4,6-C_6H_2)$_3$.

Similar correlations between the ipso-C and para-C oop distances are seen for the ambient pressure structures for the previously reported LnS_3 and **US3** structures (Supplementary Figure 14).[4,13]

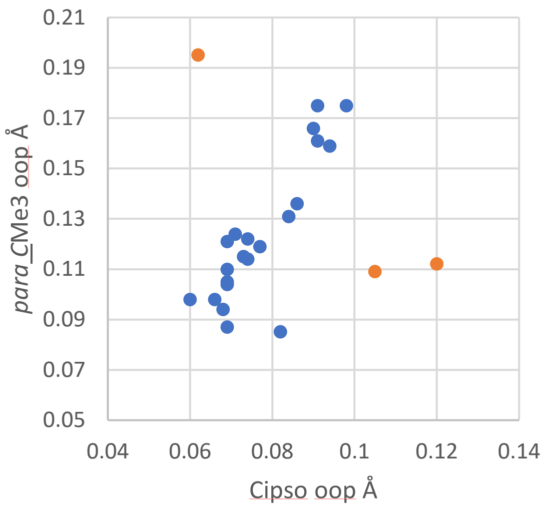

Supplementary Figure 14: The oop distances of the C_{ipso} against the corresponding para_CMe_3 oop distances for all SAr ligands in the previously reported rare earth and uranium MS3 complexes (M = (Ln = La, Ce, Pr, Nd, Sm, U).[4,13] Note that the orange outliers all correspond to SmS3. SAr = arylthiolate S-$^{t}Bu_3$-2,4,6-C_6H_2.

As the pressure is increased on the crystal of **US3** a number of weak intermolecular interactions appear to grow in, including C-H to π and π-π interactions (Tables S5 and S6 below), but these do not appear to have a significant influence on either the packing or internal structure of **US3**.

C-H	Ring	Symmetry	Pressure (GPa)	Distance (Å)
C1317-H13172	3	1-X,1-Y,-Z	0.52	3.0
C1316-H13162	3	1-X,1-Y,-Z	0.6	2.91
C1316-H13163	3	1-X,1-Y,-Z	3.0, 3.28, 3.75, 3.95, 4.45, 4.59, 4.95, 5.25	2.96, 2.94, 2.93, 2.89, 2.92, 2.90, 2.87, 2.84
C1113-H11132	2	X,Y,Z	3.0, 3.28, 3.75, 3.95, 4.20, 4.45, 4.59, 4.95, 5.25	2.55, 2.56, 2.54, 2.54, 2.52, 2.51, 2.51, 2.49, 2.48
C1112-H11122	2	1/2+X,3/2-Y,1/2+Z	3.0, 3.28, 3.75, 3.95,	2.54, 2.54, 2.52, 2.47,

			4.20, 4.45, 4.59, 4.90, 5.25	2.50, 2.46, 2.46, 2.43, 2.43
C1112-H11121	2	1/2+X,3/2-Y,1/2+Z	3.95, 5.25	2.99, 2.99
C1217-H12172	1	-1/2+X,3/2-Y,-1/2+Z	3.95	2.71

Supplementary Table 5: C-H – π interactions for US3 at various pressures. US3 = U(S-^{t}Bu$_3$-2,4,6-C_6H_2)$_3$. H-atom positions normalised.

**Ring**	**Ring**	**Symmetry**	**Pressure (GPa)**	**Distance (Å)**
3	3	1-X,1-Y,-Z	3.0, 3.95, 4.20, 4.45, 4.59, 4.90, 5.25	5.977(9), 5.940(9), 5.989(7), 5.965(7), 5.955(3), 5.995(5), 5.932(4)
1	2	1/2+X,3/2-Y,1/2+Z	3.95	5.574(6)
2	1	-1/2+X,3/2-Y,-1/2+Z	3.95	5.574(6)
3	2	X, Y, Z	4.90, 5.25	5.930(4), 5.975(5)

Supplementary Table 6: Ring-ring interactions for US3 at various pressures. US3 = U(S-^{t}Bu$_3$-2,4,6-C_6H_2)$_3$.

Volume Change of UN3 and US3 with Pressure

The molecular volumes (isodensity surface of 0.001 electrons/Bohr^3) of **UN3** and **US3m** were determined for the optimized pyramidal and transition state structures. For **UN3**, there is a small increase, 1% between the pyramidal and planar forms. Experimentally, the volume defined by the van der Waals surface of the pyramidal molecules of **UN3** in the experimental crystal structures shows a modest reduction between ambient pressure and 4.80 GPa (Fig. S15), but a systematic trend is rather difficult to discern, with most points scattered within 0.5% of the ambient pressure volume. The combination of the computational and experimental data implies that the molecular volume of the pyramidal form of **UN3** is quite insensitive to pressure, but would increase slightly on becoming planar. A planar form of **UN3** would therefore not be promoted by application of pressure.

The molecular volume of **US3** in the experimental crystal structures shows a linear decrease between ambient pressure and 5.25 GPa, with a 2% reduction in volume as the molecule changes from a pyramidal to a planar geometry (Fig. S15). The volume reduction in the theoretical structures is still more marked, with the volume of the 0.001 electrons/Bohr^3 isosurface of **US3m** being 7% smaller in the planar than in the pyramidal form. The planar form of **US3** is therefore stabilised by increased pressure.

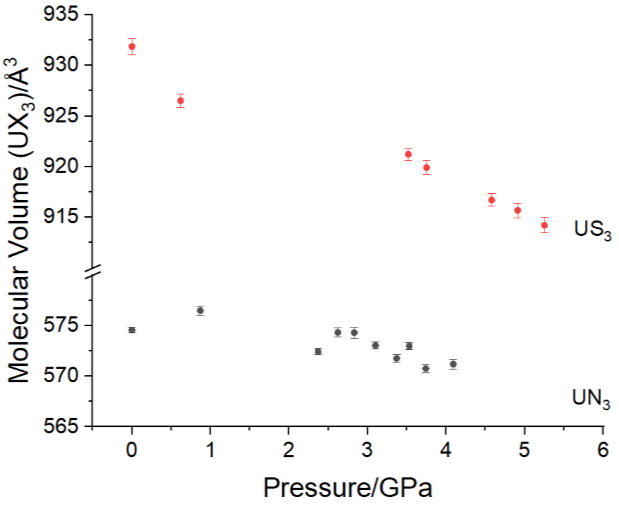

Supplementary Figure 15: Experimentally observed change in volume of US3 and UN3 with pressure. Error bars show the standard uncertainty in the measurements. **UN3** = $U(N(SiMe_3)_2)_3$, **US3** = $U(S\text{-}^tBu_3\text{-}2,4,6\text{-}C_6H_2)_3$.

Computational details and analysis

The Gaussian 16 software package, revision C.01, was used for all density functional theory calculations.[14] The hybrid density functional approximation, PBE0, was used,[15, 16] with dispersion treated by Grimme's D3 correction and the Becke-Johnson damping parameters (D3-BJ).[17-21] Dunning's correlation-consistent basis sets[22-25] of polarized triple-ζ quality were used for all atoms, except H for which the polarized double-ζ basis set was used, and uranium, which was treated with Stuttgart-Bonn small-core (60 electrons) relativistic effective core potential, in combination with the associated segmented valence basis set.[26] An ultrafine integration grid was used. Molecular volumes were computed using the "volume" keyword. The Natural Bond Orbital (NBO 7.0) software package was used to analyse the electronic structures produced by the density functional theory calculations, as discussed below.[27] The QTAIM bonding analysis was computed with the AIMAll software package, using .wfx files produced by Gaussian 16.[28]

Second order perturbation theory analysis has been carried out within the NBO framework on **US3m** and **UN3** in both pyramidal and transition state forms, focusing on the α spin NBOs. For pyramidal **US3m**, there are 1031 donor-acceptor interactions larger than the default cut off of 0.25 kcal mol^{-1}. Focusing on those above 5 kcal mol^{-1} (chosen as the only number quoted by Roger *et al.*,[4] we find

- 5 donations from agostic C-H of tBu to empty U d/f (totalling 47.5 kcal mol^{-1}). [Note that 4 of the first 5 interactions below 5 kcal mol^{-1} are also agostic C-H of tBu to empty U d/f (totalling 18.3 kcal mol^{-1})].
- 1 donation from a C_{ipso}-C_{ortho} bond to empty U f/s (7.0 kcal mol^{-1}).

- 3 donations from U-S bonds to C_{ipso}-C_{ortho} π^* (totalling 23.4 kcal mol^{-1}).
- The other interactions above 5 kcal mol^{-1} are donations from C-C π bonding NBOs to C-C π^* NBOs (totalling 190.7 kcal mol^{-1}).

For the **US3m** transition state structure, there are 963 interactions above 5 kcal mol^{-1}, comprising

- 5 donations from agostic C-H of tBu to empty U d/f (totalling 38.1 kcal mol^{-1}).
- 3 donations from U-S bonds to C_{ipso}-C_{ortho} π^* (totalling 22.0 kcal mol^{-1}).
- The other interactions above 5 kcal mol^{-1} are donations from C-C π bonding NBOs to C-C π^* NBOs (totalling 192.2 kcal/mol).

Note that the interactions directly below 5 kcal mol^{-1} cut off are not, unlike the pyramidal form, agostic C-H of tBu to empty U d/f, but 1 is donation from C_{ipso}-C_{ortho} to empty U f/s (3.7 kcal mol^{-1}).

Thus, comparison of pyramidal with the transition state structures of **US3m** indicates

- C-C π bonding NBOs to C-C π^* NBOs interactions are about the same (190.7 vs 192.2 kcal mol^{-1}).
- donation from U-S bonds to C_{ipso}-C_{ortho} π^* is about the same (23.4 vs 22.0 kcal mol^{-1}).
- C_{ipso}-C_{ortho} bond to empty U f/s is minor in both cases and a little reduced in the transition state (7.0 to 3.7 kcal mol^{-1}).
- agostic C-H of tBu to empty U d/f is much reduced (47.5 vs 38.1 kcal mol^{-1} if we focus on interactions above 5 kcal mol^{-1}, but note also that the additional 18.3 kcal mol^{-1} for interactions just below kcal mol^{-1} in the pyramidal structure isn't there in the transition state).

For **UN3** a rather different picture emerges. For the pyramidal structure, there are only two interactions larger than 5 kcal mol^{-1} in the α spin NBO manifold (*vs* 26 for **US3m**). These are both donations from agostic C-H to empty U (totalling 10.3 kcal mol^{-1}). Below 5 kcal mol^{-1}, 9 of the first 11 interactions are donations from U-N to Si-C* (totalling 33.4 kcal mol^{-1}). The other two are donations from N-Si to U (totalling 7.2 kcal mol^{-1}).

For the transition state structure, there are now 6 interactions larger than 5 kcal mol^{-1} in the α spin NBO manifold (*vs* 25 for **US3m**). Two are donations from agostic C-H to empty U (totalling 13.7 kcal mol^{-1}). Two are donations from N-Si to U (totalling 11.0 kcal mol^{-1}). Two are donations from Si-C to U (totalling 10.9 kcal mol^{-1}). Below 5 kcal mol^{-1}, the first 9 U-N to Si-C* interactions total 34.0 kcal mol^{-1}.

Comparison of pyramidal with planar **UN3** thus indicates slightly stronger interactions in the latter, by contrast to **US3m**, though this must be seen in the context of generally much weaker stabilising interactions in **UN3** *vs* **US3m**.

To assess the metal-ligand bonding in **UN3** and **US3m** we have analysed the composition of the Natural Localised Molecular Orbitals (NLMOs). Use of the NBO CHOOSE option to define each U-X bond as double produced one σ-type and one π-type NBO (and hence NLMO) per U-N, whereas for **US3m** the U-S bonding is solely π-type within the NBO framework. The lack of symmetry precludes rigorous separation into σ and π MOs, and hence we use the designations "σ-type" and "π-type" identifying orbitals by their principal character. As the early actinides primarily use 6d rather than 5f orbitals for metal-ligand bonding, the contribution of the uranium d orbitals ([%M × d]) in the σ- and π-type U-X NLMOs of **UN3** and **US3m** were calculated and are summarised in Table S7. In both pyramidal and planar forms of **UN3**, there is modest U d contribution to both σ-type and π-type NLMOs, with the contribution in the former being slightly larger. The d character of the U-S NLMOs is substantially larger, suggestive of greater covalency.

	%M × d character in U-X NLMO			
	Optimized (pyramidal)		Transition State (planar or near-planar)	
System	σ-type	π-type	σ-type	π-type
UN3	6.2	4.5	5.8	3.9
US3m		18.6		18.1

Supplementary Table 7: The calculated uranium d-character in the U-X NLMOs of UN3 and US3m.

Table S8 presents the Wiberg bond index (a NBO-computed measure of the M-X bond order) and NLMO composition metric [%U × Overlap], and the QTAIM bond critical point (BCP) electron density ρ_{BCP} and delocalisation (bond order) index δ(M,X). The two bond order metrics are consistent with single bonds, although while the Wiberg approach indicates larger bond orders in the sulfur system, the opposite is true for δ(U,X). The BCP electron densities are also larger for the U-N bonds, though as these and other QTAIM metrics are highly M-X distance dependent, it is not surprising that ρ_{BCP} and δ(U,X) are smaller for the sulfur system. The NLMO [%M × Overlap] bond metric agrees with the Wiberg and U d character data in finding the U-S bond to be the most covalent.

	**System**	**Wiberg Bond Index**	**NLMO: [%M × Overlap]**	**ρ_{BCP}**	**δ(U,X)**
**UN3**	**Optimized (pyramidal)**	0.62	4.6	0.098	0.72
	**Transition State (planar at U)**	0.60	4.3	0.093	0.70
**US3m**	**Optimized (pyramidal)**	0.89	10.7	0.065	0.62
	**Transition State (near-planar at U)**	0.85	10.4	0.064	0.62

Supplementary Table 8: U-X bond metrics for the pyramidal and transition state structures of UN3 and US3m.

Most pertinent to this study, there are only very small changes in all of the data in Tables S7 and S8 between the pyramidal and planar forms of both systems. These changes evidence very modest weakening of the uranium-ligand bond in the planar TS, but the differences in the high-pressure behaviour of **UN3** and **US3m** are very unlikely to be driven by such small changes in the metal-ligand bonding.

Optimised Cartesian Co-ordinates (Å)

UN3

U	-0.000118	-0.000150	0.400078
N	1.478641	1.661790	-0.113422
Si	2.723958	1.588284	-1.311347
C	2.315733	0.203457	-2.519446
C	4.407951	1.233978	-0.547059
C	2.859502	3.190565	-2.289209
H	3.122345	0.088317	-3.251943
H	2.186535	-0.759055	-2.011265
H	1.397430	0.427368	-3.073818
H	5.161061	1.059581	-1.323876
H	4.373734	0.347234	0.094070
H	4.748173	2.073747	0.066989
H	3.590240	3.086909	-3.099217
H	1.896224	3.462296	-2.733737
H	3.185737	4.021145	-1.653584
Si	1.254159	2.943699	1.013250
C	0.016086	2.332661	2.334758
C	0.529555	4.501798	0.258233
C	2.820688	3.404254	1.944554
H	-0.112204	3.133391	3.070596
H	-0.992209	2.121081	1.948064
H	0.383490	1.469507	2.910922
H	0.282808	5.238462	1.031083
H	-0.380365	4.281156	-0.307555
H	1.243981	4.963273	-0.430601
H	2.605364	4.136558	2.730788
H	3.278337	2.524922	2.409992
H	3.560139	3.849090	1.270228
N	-2.179147	0.449194	-0.113313
Si	-2.736490	1.564652	-1.311670
C	-1.331621	1.903828	-2.517975
C	-3.271864	3.200449	-0.548028
C	-4.190556	0.881469	-2.291775
H	-1.635580	2.658298	-3.252052
H	-0.434796	2.275552	-2.009234
H	-1.064102	0.996297	-3.070840
H	-3.497409	3.939458	-1.325235
H	-2.486334	3.614247	0.092499
H	-4.169086	3.076078	0.066392
H	-4.465340	1.566718	-3.101546
H	-3.943591	-0.088265	-2.736727
H	-5.073644	0.748014	-1.657217
Si	-3.178105	-0.385896	1.012412
C	-2.031781	-1.153302	2.335140
C	-4.164511	-1.792188	0.256106
C	-4.360779	0.740872	1.942561
H	-2.662214	-1.664317	3.070342
H	-1.344874	-1.921252	1.948748
H	-1.467596	-0.404308	2.911962
H	-4.679380	-2.374956	1.028168
H	-3.517606	-2.469177	-0.309579
H	-4.920973	-1.403972	-0.433112
H	-4.888230	0.188293	2.728226
H	-3.828295	1.576639	2.408581
H	-5.115160	1.159078	1.267658
N	0.700454	-2.111469	-0.113493
Si	0.013929	-3.152229	-1.311999
C	-0.982091	-2.105322	-2.518436
C	-1.133875	-4.434634	-0.548207
C	1.333323	-4.069176	-2.291928
H	-1.484755	-2.745740	-3.251684
H	-1.751129	-1.512950	-2.009545
H	-0.329171	-1.421293	-3.072226
H	-1.660207	-5.000619	-1.325173
H	-1.885619	-3.962073	0.092185
H	-0.576320	-5.148231	0.066529
H	0.877820	-4.650157	-3.101740
H	2.049345	-3.370064	-2.736792
H	1.890718	-4.766843	-1.657179
Si	1.922545	-2.558626	1.013093
C	2.013176	-1.180908	2.334389
C	3.633927	-2.710381	0.257566
C	1.538124	-4.145501	1.944484
H	2.770509	-1.470712	3.070296
H	2.334568	-0.202409	1.946967
H	1.082126	-1.066651	2.910716
H	4.395563	-2.865320	1.030060
H	3.897457	-1.811720	-0.307941
H	3.676051	-3.559516	-0.431721
H	2.280350	-4.325203	2.730376
H	0.547932	-4.102254	2.410197
H	1.553527	-5.008327	1.270207

UN3 – transition state

U	0.000145	-0.061218	-0.000994
N	-2.057182	-1.098197	0.004890

Si	-3.237280	-0.615059	-1.145629
C	-2.373809	0.572866	-2.339614
C	-4.690917	0.277596	-0.355846
C	-3.905066	-2.055258	-2.154809
H	-3.089802	0.967659	-3.068316
H	-1.930835	1.439106	-1.830013
H	-1.592378	0.060742	-2.916445
H	-5.380117	0.666813	-1.113636
H	-4.350860	1.117273	0.258753
H	-5.253857	-0.401228	0.293537
H	-4.578643	-1.704419	-2.944745
H	-3.089644	-2.615388	-2.625057
H	-4.468372	-2.749847	-1.521868
Si	-2.123603	-2.334879	1.189888
C	-0.584032	-2.102545	2.284290
C	-2.051252	-4.066416	0.462553
C	-3.624554	-2.217164	2.314059
H	-0.533054	-2.934810	2.994394
H	0.378441	-2.131340	1.747326
H	-0.638284	-1.188481	2.889465
H	-1.999748	-4.828251	1.248498
H	-1.175682	-4.184922	-0.184115
H	-2.940521	-4.267850	-0.143720
H	-3.572567	-2.954644	3.122798
H	-3.701455	-1.220945	2.762189
H	-4.545783	-2.404107	1.751381
N	2.062800	-1.087742	-0.003781
Si	2.135644	-2.323451	-1.189563
C	0.594411	-2.098612	-2.283174
C	2.072741	-4.055997	-0.463832
C	3.635341	-2.196264	-2.314319
H	0.548321	-2.930008	-2.994606
H	-0.367782	-2.134052	-1.746113
H	0.643222	-1.183385	-2.887042
H	2.021826	-4.817117	-1.250515
H	1.199539	-4.178533	0.185282
H	2.964590	-4.254776	0.139510
H	3.586576	-2.932212	-3.124648
H	3.707089	-1.198673	-2.760245
H	4.557758	-2.379885	-1.752512
Si	3.240459	-0.599889	1.147373
C	2.372373	0.585606	2.340256
C	4.691640	0.297557	0.358397
C	3.912158	-2.038182	2.156589
H	3.086309	0.982154	3.070035
H	1.927991	1.450537	1.829748
H	1.591159	0.071841	2.915968
H	5.379017	0.688774	1.116815
H	4.349344	1.136342	-0.256219
H	5.257260	-0.379254	-0.290745
H	4.584579	-1.686011	2.946904
H	3.098043	-2.600690	2.626252
H	4.477376	-2.731023	1.523434
N	-0.005663	2.228773	0.001001
Si	0.971138	3.000107	-1.189008
C	1.506691	1.664130	-2.419790
C	2.524056	3.777513	-0.469670
C	0.039075	4.317680	-2.153547
H	2.144027	2.106580	-3.193128
H	2.094945	0.863403	-1.951003
H	0.645923	1.225637	-2.941477
H	3.173004	4.167270	-1.262086
H	3.097821	3.047535	0.110114
H	2.272632	4.607710	0.198018
H	0.655581	4.714557	-2.967875
H	-0.880301	3.910433	-2.587437
H	-0.237004	5.157257	-1.506317
Si	-0.986370	2.996838	1.189961
C	-1.517391	1.659121	2.420551
C	-2.542104	3.767319	0.469077
C	-0.060618	4.319831	2.153217
H	-2.157923	2.098229	3.193143
H	-2.101113	0.855998	1.950152
H	-0.655327	1.224140	2.943009
H	-3.193430	4.154083	1.261012
H	-3.112115	3.034822	-0.111212
H	-2.293919	4.598697	-0.198350

H	-0.678864	4.713988	2.967552
H	0.861184	3.917963	2.586906
H	0.210531	5.160509	1.505263

US3m

U	0.046520	-0.098661	0.001439
S	2.018785	1.740923	-0.311917
C	2.852813	0.380835	-1.090370
C	2.281652	-0.198582	-2.260628
C	2.730484	-1.465586	-2.645716
C	3.763398	-2.094545	-1.991143
C	4.431101	-1.432708	-0.975051
C	4.014899	-0.197019	-0.500794
C	4.867913	0.503381	0.572959
C	4.157406	0.606076	1.927140
C	5.268736	1.898103	0.078763
C	6.172085	-0.253852	0.831056
C	1.353616	0.570688	-3.237567
C	2.044345	1.882319	-3.622804
C	-0.056512	0.881517	-2.722878
C	1.138599	-0.225424	-4.526819
S	-2.359033	0.757725	0.800002
C	-1.590721	2.369339	0.815196
C	-1.928754	3.335933	-0.179278
C	-1.081130	4.423488	-0.338395
C	0.019548	4.622698	0.474261
C	0.226340	3.786900	1.546226
C	-0.576685	2.667074	1.771062
C	-0.382798	1.904985	3.103308
C	0.230199	0.515944	2.916451
C	-1.720635	1.789890	3.838337
C	0.572465	2.651580	4.037896
C	-3.224809	3.297978	-1.011022
C	-3.189899	2.232435	-2.107427
C	-4.427824	3.082552	-0.084691
C	-3.473618	4.633242	-1.717504
S	-0.380280	-2.455122	-1.276452
C	-1.349839	-2.684632	0.196432
C	-0.682623	-2.802256	1.453181
C	-1.446614	-2.648266	2.616061
C	-2.806384	-2.459523	2.567068
C	-3.458142	-2.516710	1.348264
C	-2.774878	-2.652381	0.148741
C	-3.595215	-2.821127	-1.141980
C	-3.505395	-1.594486	-2.053337
C	-3.143671	-4.084767	-1.882668
C	-5.082239	-3.014048	-0.834279
C	0.779013	-3.292691	1.613726
C	1.841212	-2.297412	1.145541
C	1.108576	-3.590592	3.077808
C	0.942710	-4.606713	0.843897
H	2.268283	-1.968357	-3.482760
H	5.294749	-1.911956	-0.535347
H	4.836662	1.056742	2.659217
H	3.262098	1.223423	1.863163
H	3.882352	-0.387375	2.296808
H	5.910750	2.381455	0.823200
H	5.831525	1.820972	-0.857284
H	4.401230	2.536667	-0.090745
H	6.774170	0.322652	1.539240
H	6.762470	-0.380247	-0.081637
H	6.001121	-1.239454	1.276119
H	1.435482	2.419266	-4.358352
H	3.021350	1.675654	-4.070088
H	2.187800	2.528757	-2.754980
H	-0.663578	1.285430	-3.536460
H	-0.062610	1.652843	-1.941066
H	-0.589506	-0.043920	-2.434320
H	0.559003	0.388061	-5.222481
H	2.084902	-0.475975	-5.013066
H	0.575508	-1.148418	-4.353597
H	-1.283937	5.150047	-1.112704
H	1.041424	4.007948	2.219529
H	0.505025	0.079143	3.882134
H	1.166505	0.594370	2.342627
H	-0.492634	-0.187259	2.477503
H	-1.570798	1.288183	4.800732

H	-2.127919	2.786794	4.032850
H	-2.452561	1.222311	3.262017
H	0.599230	2.127532	4.997829
H	0.239402	3.675854	4.225566
H	1.596268	2.681116	3.651744
H	-4.159778	2.194069	-2.615722
H	-2.432370	2.486704	-2.854461
H	-2.974261	1.243437	-1.707552
H	-5.351811	3.138897	-0.670144
H	-4.461812	3.865229	0.680493
H	-4.398579	2.114336	0.414558
H	-4.442907	4.581600	-2.222167
H	-3.506443	5.470765	-1.013723
H	-2.722477	4.848817	-2.483561
H	-0.965879	-2.687967	3.583124
H	-4.535957	-2.440816	1.342197
H	-4.159226	-1.737411	-2.920954
H	-3.838402	-0.701759	-1.517131
H	-2.490071	-1.428859	-2.411434
H	-3.762531	-4.224296	-2.775580
H	-2.101079	-4.028288	-2.195812
H	-3.267154	-4.965276	-1.243119
H	-5.605455	-3.217253	-1.773057
H	-5.535865	-2.119352	-0.396743
H	-5.258312	-3.861602	-0.164408
H	2.843058	-2.678186	1.363549
H	1.775806	-1.350284	1.701359
H	1.816157	-2.138828	0.063175
H	2.119716	-4.004667	3.127443
H	1.094088	-2.689955	3.700809
H	0.425490	-4.325785	3.511383
H	1.956865	-4.995031	0.987955
H	0.775079	-4.467963	-0.225171
H	0.232653	-5.351752	1.215665
H	4.081009	-3.084676	-2.299285
H	0.684067	5.461623	0.299697
H	-3.371151	-2.309131	3.480578

US3m – transition state

U	0.085536	0.030329	-0.182354
S	-0.101433	-2.607036	0.370134
C	-1.453957	-2.428214	-0.764550
C	-1.180520	-2.088731	-2.121845
C	-2.236596	-1.600571	-2.896976
C	-3.515059	-1.510833	-2.397592
C	-3.793450	-2.015207	-1.139032
C	-2.800964	-2.509548	-0.305699
C	-3.212787	-3.225048	0.994818
C	-2.764402	-2.510746	2.273722
C	-2.644692	-4.649357	0.968902
C	-4.733334	-3.359974	1.093576
C	0.159081	-2.409158	-2.831395
C	0.494739	-3.886358	-2.605837
C	1.346886	-1.548378	-2.390661
C	0.045149	-2.200569	-4.342663
S	2.265306	1.390619	0.676919
C	2.787897	-0.218787	1.229033
C	3.848779	-0.900457	0.561070
C	3.999365	-2.257700	0.808535
C	3.207442	-2.930893	1.721441
C	2.308170	-2.226648	2.486409
C	2.085933	-0.861958	2.288857
C	1.200062	-0.120412	3.318984
C	-0.117980	0.407711	2.744391
C	1.992091	1.039106	3.929923
C	0.797091	-1.042498	4.471848
C	4.899894	-0.197775	-0.319870
C	4.346442	0.304302	-1.655972
C	5.513077	0.966614	0.467654
C	6.056154	-1.138887	-0.665428
S	-1.993844	1.292593	-1.361897
C	-1.380526	2.633165	-0.358444
C	-2.011701	2.943521	0.882966
C	-1.331097	3.773114	1.767371
C	-0.106080	4.328636	1.453679
C	0.414926	4.154039	0.191299
C	-0.211665	3.346699	-0.758017
C	0.307919	3.406753	-2.212393
C	0.859769	2.081005	-2.741713
C	-0.834738	3.886832	-3.113381
C	1.449111	4.415737	-2.354228
C	-3.439720	2.499367	1.253776
C	-3.547257	0.998259	1.528960
C	-3.930289	3.199725	2.523088
C	-4.405824	2.908142	0.135790
H	-2.056833	-1.281778	-3.913670
H	-4.821913	-2.016904	-0.806008
H	-3.163964	-3.046696	3.141848
H	-1.679271	-2.480770	2.358383
H	-3.153229	-1.489982	2.304455
H	-2.970837	-5.192087	1.862810
H	-3.009217	-5.190024	0.089330
H	-1.553726	-4.650141	0.948386
H	-4.973431	-3.942836	1.987453
H	-5.158392	-3.882798	0.231431
H	-5.228726	-2.388686	1.193577
H	1.407745	-4.146728	-3.152457
H	-0.319264	-4.516605	-2.976410
H	0.650871	-4.105522	-1.548311
H	2.226510	-1.790802	-2.993791
H	1.645365	-1.738914	-1.351104
H	1.165037	-0.481777	-2.588334
H	0.982958	-2.514319	-4.810308
H	-0.760490	-2.799272	-4.775649
H	-0.118896	-1.150882	-4.607587
H	4.763216	-2.815592	0.285292
H	1.754607	-2.757139	3.247001
H	-0.749811	0.807990	3.542612
H	-0.698490	-0.415980	2.297359
H	0.044155	1.254841	2.062850
H	1.386632	1.538318	4.694427
H	2.901805	0.660858	4.406106
H	2.274289	1.776479	3.176887
H	0.242418	-0.455162	5.209416
H	1.668835	-1.470382	4.974039
H	0.145428	-1.858200	4.143060
H	5.155230	0.767918	-2.231546
H	3.952234	-0.527491	-2.247179
H	3.560593	1.043557	-1.514053
H	6.304844	1.435111	-0.127002
H	5.957129	0.602368	1.399858
H	4.772512	1.728879	0.712196
H	6.809709	-0.572622	-1.220506
H	6.537213	-1.546460	0.228843
H	5.740106	-1.971089	-1.302642
H	-1.763311	3.996305	2.732632
H	1.337055	4.658139	-0.057926
H	1.333309	2.235074	-3.717457
H	1.641801	1.700557	-2.070966
H	0.062254	1.347216	-2.874560
H	-0.480933	3.971426	-4.146808
H	-1.678237	3.194196	-3.093201
H	-1.184335	4.872267	-2.789764
H	1.730937	4.473928	-3.409518
H	2.338365	4.113177	-1.791590
H	1.154246	5.419136	-2.034689
H	-4.572462	0.753808	1.829860
H	-2.888314	0.717700	2.356380
H	-3.299932	0.399569	0.652364
H	-4.970255	2.909550	2.698135
H	-3.358174	2.906780	3.409445
H	-3.899531	4.289622	2.431498
H	-5.428500	2.635929	0.418750
H	-4.169324	2.415473	-0.807642
H	-4.371855	3.991718	-0.018071
H	-4.309093	-1.090640	-3.004402
H	3.328330	-3.998666	1.866733
H	0.421158	4.935825	2.181425

Supplementary References

1) M. J. Monreal, R. K. Thomson, T. Cantat, N. E. Travia, B. L. Scott, and J. L. Kiplinger, *Organometallics,* **2011**, 30, 2031–2038

2) W. Rundel, *Chem. Ber.***1968**, *101*, 2956

3) D.L. Clark, A.P. Sattelberger, R.A. Andersen, *Inorg. Synth.,* **1997**, 31, 307

4) M. Roger, N. Barros, T. Arliguie, P. Thuéry, L. Maron and M. Ephritikhine, *J. Am. Chem. Soc.* **2006**, 128, 27, 8790–8802

5) W. G. Van der Sluys, C. J. Burns, and A. P. Sattelberger *Organometallics* **1989**, 8, 3, 855–857

6) E. D. Brady, D. L. Clark, J. C. Gordon, P. J. Hay, D. W. Keogh, R. Poli, B. L. Scott, J. G. Watkin *Inorg. Chem.* **2003**, *42,* 21, 6682–6690

7) J. Gonzalez-Platas, M. Alvaro, F. Nestola and R. Angel. *EosFit7-GUI*: a new graphical user interface for equation of state calculations, analyses and teaching *J. Appl. Cryst.* **2016**. 49, 1377-1382

8) M. Roger, L. Belkhiri, P. Thuéry, T. Arliguie, M. Fourmigué, A. Boucekkine, M. Ephritikhine, *Organometallics* **2005**, *24*, 4940–4952

9) T. Arliguie, C. Lescop, L. Ventelon, P. C. Leverd, P. Thuéry, M. Nierlich, M. Ephritikhine, *Organometallics* **2001**, *20*, 3698–3703

10) E. M. Matson, P. E. Fanwick, S. C. Bart, *Organometallics* **2011**, *30*, 5753–5762

11) Daniel Pividori, Matthias E. Miehlich, Benedikt Kestel, Frank W. Heinemann, Andreas Scheurer, Michael Patzschke and Karsten Meyer *Inorg. Chem.* **2021**, 60, 21, 16455–16465

12) Carla Slebodnick, Jing Zhao, Ross Angel, Brian E. Hanson, Yang Song, Zhenxian Liu, and Russell J. Hemley, *Inorg. Chem.* **2004**, 43, 17, 5245–5252

13) B. Cetinkaya, P. B. Hitchcock, M. F. Lappert, R. G. Smith, *J. Chem. Soc. Chem. Commun.* **1992**, 932–934

14) Gaussian 16, Revision C.01, M. J. Frisch, G. W. Trucks, H. B. Schlegel, G. E. Scuseria, M. A. Robb, J. R. Cheeseman, G. Scalmani, V. Barone, G. A. Petersson, H. Nakatsuji, X. Li, M. Caricato, A. V. Marenich, J. Bloino, B. G. Janesko, R. Gomperts, B. Mennucci, H. P. Hratchian, J. V. Ortiz, A. F. Izmaylov, J. L. Sonnenberg, D. Williams-Young, F. Ding, F. Lipparini, F. Egidi, J. Goings, B. Peng, A. Petrone, T. Henderson, D. Ranasinghe, V. G. Zakrzewski, J. Gao, N. Rega, G. Zheng, W. Liang, M. Hada, M. Ehara, K. Toyota, R. Fukuda, J. Hasegawa, M. Ishida, T. Nakajima, Y. Honda, O. Kitao, H. Nakai, T. Vreven, K. Throssell, J. A. Montgomery, Jr., J. E. Peralta, F. Ogliaro, M. J. Bearpark, J. J. Heyd, E. N. Brothers, K. N. Kudin, V. N. Staroverov, T. A. Keith, R. Kobayashi, J. Normand, K. Raghavachari, A. P. Rendell, J. C. Burant, S. S. Iyengar, J. Tomasi, M. Cossi, J. M. Millam, M. Klene, C. Adamo, R. Cammi, J. W. Ochterski, R. L. Martin, K. Morokuma, O. Farkas, J. B. Foresman, and D. J. Fox, Gaussian, Inc., Wallingford CT, 2019

15) Ernzerhof M, Scuseria GE. Assessment of the Perdew–Burke–Ernzerhof exchange-correlation functional. *J Chem Phys* **1999** 110, 5029-5036

16) Toward reliable density functional methods without adjustable parameters: The PBE0 model. *J Chem Phys* **1999** 110, 6158-6170

17) Grimme S, Antony J, Ehrlich S, Krieg H. A consistent and accurate ab initio parametrization of density functional dispersion correction (DFT-D) for the 94 elements H-Pu. *J Chem Phys* **2010** 132, 154104

18) Johnson ER, Becke AD. A post-Hartree–Fock model of intermolecular interactions. *J Chem Phys* **2005** 123, 1024101

19) Johnson ER, Becke AD. A post-Hartree-Fock model of intermolecular interactions: Inclusion of higher-order corrections. *J Chem Phys* **2006** 124, 174104

20) Grimme S, Ehrlich S, Goerigk L. Effect of the damping function in dispersion corrected density functional theory. *J Comp* Chem **2011** 32, 1456-1465

21) Becke AD, Johnson ER. A density-functional model of the dispersion interaction. *J Chem Phys* **2005** 123, 154101

22) T. H. J. Dunning*, J. Chem. Phys*., **1989**, 90, 1007–1023

23) ; R. A. Kendall, T. H. Dunning and R. J. Harrison, *J. Chem. Phys.,* **1992**, 96, 6796–6806

24) D. E. Woon and T. H. Dunning, *J. Chem. Phys.,* **1993**, 98, 1358–1371

25) A. K. Wilson, T. Van Mourik and T. H. Dunning, *THEOCHEM*, **1996**, 388, 339–349

26) Cao X, Dolg M. Segmented contraction scheme for small-core actinide pseudopotential basis sets. *J Mol Struct THEOCHEM* **2004** 673, 203-209

27) NBO 7.0. E. D. Glendening, J. K. Badenhoop, A. E. Reed, J. E. Carpenter, J. A. Bohmann, C. M. Morales, P. Karafiloglou, C. R. Landis, and F. Weinhold, Theoretical Chemistry Institute, University of Wisconsin, Madison, WI **2018**

28) Keith TA. AIMALL 19.02.13 Gristmill Software, Overland Park KS, USA. **2019**
